# A rapid model for developing dry surface biofilms of *Staphylococcus aureus* and *Pseudomonas aeruginosa* for in vitro disinfectant efficacy testing

**DOI:** 10.1186/s13756-020-00792-9

**Published:** 2020-08-17

**Authors:** Carine A. Nkemngong, Maxwell G. Voorn, Xiaobao Li, Peter J. Teska, Haley F. Oliver

**Affiliations:** 1 Department of Food Science, Purdue University, 745 Agriculture Mall Drive, West Lafayette, IN 47907 USA; 2 Diversey Inc., Charlotte, NC 28273 USA

**Keywords:** Dry surface biofilms, *Staphylococcus aureus*, *Pseudomonas aeruginosa*, Disinfectant efficacy testing

## Abstract

**Background:**

Bacterial biofilms persistent on dry environmental surfaces in healthcare facilities play an important role in the occurrence of healthcare associated infections (HAI). Compared to wet surface biofilms and planktonic bacteria, dry surface biofilms (DSB) are more tolerant to disinfection. However, there is no official method for developing DSB for in vitro disinfectant efficacy testing. The objectives of this study were to (i) develop an in vitro model of DSB of *S. aureus* and *P. aeruginosa* for disinfectant efficacy testing and (ii) investigate the effect of drying times and temperatures on DSB development. We hypothesized that a minimum six log_10_ density of DSB could be achieved on glass coupons by desiccating wet surface biofilms near room temperatures. We also hypothesized that a DSB produced by the model in this study will be encased in extracellular polymeric substances (EPS).

**Methods:**

*S. aureus* ATCC-6538 and *P. aeruginosa* ATCC-15442 wet surface biofilms were grown on glass coupons following EPA MLB SOP MB-19. A DSB model was developed by drying coupons in an incubator and viable bacteria were recovered following a modified version of EPA MLB SOP MB-20. Scanning electron microscopy was used to confirm the EPS presence on DSB.

**Results:**

Overall, a minimum of six mean log_10_ densities of DSB for disinfectant efficacy were recovered per coupon after drying at different temperatures and drying times. Regardless of strain, temperature and dry time, 86% of coupons with DSB were confirmed to have EPS.

**Conclusion:**

A rapid model for developing DSB with characteristic EPS was developed for disinfectant efficacy testing against DSB.

## Background

Healthcare Associated Infections (HAIs) are a major cause of morbidity and mortality in the United States [1, 2]. HAIs were estimated to cause about 721,800 infections in acute care hospitals in 2011, and approximately 75,000 deaths each year [3]. On average, one out of 31 hospital patients in the US contracts at least one HAI daily [4]. Although it is estimated that 30–35% of HAIs can be prevented [5], the occurrence of HAIs is still one of the leading causes of death in the US [6].

*Staphylococcus aureus* and *Pseudomonas aeruginosa* are some of the most common causes of HAIs [7, 8]. These pathogens can grow in the environment as biofilms [9, 10]. They attach to inert surfaces and can be encased by extracellular polymeric substances (EPS) [9, 11, 12]. Biofilms on high touch surfaces are a known contributor to HAI infections [13]; they are estimated to account for 65 and 80% of microbial and chronic infections, respectively [14].

Environmental surfaces (e.g., food trolleys, hospital commodes, bed rails) [15] and mobile patient care equipment such as blood pressure cuffs [16] and intravenous poles [17] support the formation of biofilms by providing a platform for the attachment of planktonic bacterial cells [9, 18, 19]. On any surface, biofilms are generally not evenly distributed [20] and knowledge of the factors that influence the production of EPS by bacteria as *S. aureus* under low shear conditions is limited [21]. Once formed, biofilms are capable of growing on dry surfaces in healthcare facilities for protracted periods, surviving for as long as one year [22, 23]. The definition of a DSB is controversial [24, 25] as there is no clear definition or test method to differentiate varying degrees of dehydrated or dry biofilms. Dry surface biofilms (DSB) have been reported to persistently grow on contaminated environmental surfaces in healthcare facilities and can therefore play an important role in HAIs [24]. Although dehydrated, DSB can still be characterized by the presence of EPS [20, 26]. At maturity, bacterial cells in *P. aeruginosa* [27] and *S. aureus* [28] wet surface biofilms become motile, allowing for easy dispersal and re-attachment on other surfaces [9, 13, 18]. More recently, a 2018 study by Chowdhury et al. demonstrated that DSB of *S. aureus* can be dispersed to other surfaces even though they are dehydrated [24]. However, the dispersal rates for DSB are relatively lower compared to the dispersal of planktonic bacterial cells [24]. The use of antimicrobials to disinfect contaminated surfaces in healthcare facilities plays an important role in preventing HAIs [13, 29, 30]. Studies have shown that wet surface bacterial biofilms can be more tolerant to disinfectants than planktonic cells [31–33]. In an extensive review by Otter et al., mature wet surface biofilms were estimated to be on average 1000 times less susceptible to disinfectants than planktonic bacteria [34]. The tolerance to antimicrobial disinfectants has been principally associated with EPS, which can shield underlying cells from direct contact with antimicrobials [35, 36]. Compared to wet surface biofilms, dry surface bacterial biofilms can be more tolerant to disinfection procedures [26, 37]. However, while there is an official method for testing the bactericidal efficacy of disinfectants against wet surface biofilms [38], a comparable method for developing DSB for disinfectant efficacy testing in vitro has not been extensively developed. There are very limited studies that have developed a rapid in vitro DBS biofilm model and there is no clear standard for how many days or the conditions for a wet surface biofilm to dehydrate and become a DSB. Previous DSB development models include a dehydration step [20]. Following ASTM E-2197 [39], a two-hour drying time has been used to dehydrate wet surface biofilms for disinfectant efficacy testing [45]. A previously developed model required 12 days of repeated hydration and dehydration of bacterial cells to develop DSB [20]. The objective of this study was to develop an in vitro model of *S. aureus* and *P. aeruginosa* DSB for disinfectant efficacy testing. In this study, we investigated the effect of different drying times and temperatures on the development of DSB of *S. aureus* and *P. aeruginosa.* We hypothesized that we would achieve a minimum six log_10_ bacterial density on DSB per test coupon for disinfectant efficacy testing by subjecting wet surface biofilms to desiccation at near room temperatures. We also hypothesized that DSB of *S. aureus* and *P. aeruginosa* produced by the model in this study will be encased in EPS similar to those of wet surface biofilms to mimic associated disinfection challenges.

## Methods

### Bacteria strains used in this study

*S. aureus* ATTC-6538 and *P. aeruginosa* ATCC-15442 biofilms were developed on borosilicate glass coupons (1.27 ± 0.01 cm; Biosurface Tech, Inc.). These are standard strains for EPA disinfectant efficacy testing on wet surface biofilms and for disinfectant registration for use in healthcare facilities [40].

### Development of DSB

Wet surface biofilms were established on coupons for 48 h following EPA MLB SOP MB-19-for growing biofilms using a CDC biofilm reactor [38]. Wet surface biofilms were grown in two phases (batch and continuous) as defined in the aforementioned protocol. For the batch phase, one mL of *S. aureus* or *P. aeruginosa* overnight culture were transferred into 500 mL tryptic soy broth (TSB; Becton, Dickinson and Company, Sparks, MD) batch culture medium (three TSB/L for *S. aureus* and 300 mg TSB/L for *P. aeruginosa*) in a CDC biofilm reactor (Biosurfaces Technologies, Inc., Bozeman, MT). The CDC biofilm reactor was mounted on a magnetic hot plate stirrer (Talbays, Thorofare, NJ) to initiate the growth of biofilms for 24 ± 2 h. The hot plate stirrer was set at a rotational speed of 60 ± 5 rpm at 36 ± 1 °C for *S. aureus.* The rotational speed and hot plate stirrer temperature for *P. aeruginosa* was 125 ± 5 rpm and 21 ± 2 °C, respectively, following EPA MLB SOP MB-19. The Continuous Stir Tank Reactor (CSTR) phase immediately followed the batch phase and involved a continuous flow of CSTR medium for 24 ± 2 h. A total of 500 mL or 50 mL of CSTR medium was added to 20 L of sterile distilled water in a carboy for *S. aureus* or *P. aeruginosa* respectively. This resulted in a final growth medium concentration of 1 g/L TSB for *S. aureus* and 100 mg/L TSB for *P. aeruginosa*. The 20 L CSTR growth medium in the carboys were preheated to 36 ± 1 °C *S. aureus* and maintained at 21 °C for *P. aeruginosa* before continuously pumping the medium through the CDC biofilm reactor for 24 ± 2 h. The CSTR growth medium was pumped (Cole-Palmer, Barrington, IL) at a rate of 30 ± 2 min residence time for *S. aureus* and *P. aeruginosa*.

Polypropylene rods holding coupons were rinsed in 25 ml phosphate buffered saline (PBS) for 30 s to remove planktonic bacteria cells following EPA MLB SOP MB-20 [40] before drying. Coupons with wet surface biofilms of *S. aureus* and *P. aeruginosa* were continuously dried in a standard laboratory incubator to develop DSB while still contained to the rod. The dry times for both *S. aureus* and *P. aeruginosa* coupons were 24 h, 48 h, 72 h, 96 h, and 120 h. Bacteria recovery from DSB was done within 120 h as the CDC recommends routine disinfection of healthcare surfaces at least daily or every other day (approximately 48 h) [41]. Drying temperatures were 25 °C and 30 °C for *S. aureus* to mimic near and elevated room temperatures and 16 °C and 21 °C as guided by preliminary studies conducted by our research group to establish dry surface *P. aeruginosa* biofilms*.* Incubators were disinfected with 70% ethanol prior to drying coupons. Incubator doors were also kept closed during drying.

### Recovery of viable *S. aureus* and *P. aeruginosa* cells from DSB

Bacterial cells from DSB were recovered following a modified version of EPA MLB SOP MB-20 for testing the efficacy of disinfectants against bacterial biofilms [40]. At every dry temperature (25 °C and 30 °C for *S. aureus*; 16 °C and 21 °C for *P. aeruginosa*) and at each dry time (24–120 h), three coupons with DSB (technical replicates; *N* = 15 coupons per dry temperature and bacterial strain) were harvested from the rods in the incubator and transferred into three 50 mL sterile conical tubes (Corning Science, Mexico). Coupons were harvested successively at 24 h, 48 h, 72 h, 96 h and 120 h and processed to establish mean log_10_ bacterial densities per coupon. Three control coupons with wet surface biofilms were also processed to detect viable bacteria from biofilms before dehydration. A control coupon was also harvested before drying, at each dry time, and at each drying temperature for scanning electron microscopy (SEM) imaging.

Viable bacterial cells were recovered from coupons in 50 mL sterile conical tubes with four mL of PBS. After one min, 36 mL of neutralizing buffer (1 L H_2_O + 5.2 g Difco neutralizing buffer; Becton, Dickinson and Company Sparks, MD) was added. Bacterial biofilms were released from coupons into solution through alternate cycles of vortexing and sonication [40]. The contents of the 50 mL tubes were vigorously vortexed through three one-minute cycles alternated with sonication at 45 Khz (ultra-sonic water bath; Cole-Parmer Instrument Company, Chicago, IL). All coupons were subjected to four sonication cycles (one min, one min, one min, and two min) lasting a total of five min. Viable bacteria cells of *S. aureus* and *P. aeruginosa* from coupons were quantified after serial dilution with PBS, and ten mL aliquots vacuum-filtered onto sterile filter membranes (0.2 μm pore; Pall Corporation, Port Washington, NY) and incubated following EPA MLB SOP MB-20.Five biological replicates were conducted for each dry temperature. Each batch of three control coupons with wet surface biofilms and 15 technical replicates with DSB (*N* = 18 coupons) made up one biological replicate.

### Scanning Electron microscopy

Coupons for SEM imaging were stored in an aldehyde fixative (2.5% glutaraldehyde + 0.1 M cacodylate buffer, Electron Microscopy Sciences; Hatfield, PA) at 4 °C before sample preparation for imaging. Bacteria cells on control coupons (wet surface biofilms) and DSB coupons were fixed for SEM imaging to confirm the presence of *S. aureus* and *P. aeruginosa* extracellular matrices typical of bacterial biofilms. Harvested coupons were placed in 24 well plates (Corning Science, Mexico) and fixed for SEM imaging following the step-wise sample fixation procedure used by the Life Sciences Core facility at Purdue University and as described by Murtey and Ramasamy [42] with modifications. Following glutaraldehyde fixation, each coupon was rinsed three times with one mL of distilled water. After, the third rinse, each coupon was fixed with one mL of 1% osmium tetroxide for a contact time of 30 min per coupon. Osmium tetroxide was removed and each coupon rinsed three times with one mL distilled water. Each sample was progressively fixed 50% ethanol, 75% ethanol, 95% ethanol and 100% ethanol, with each individual alcohol treatment lasting 10 min. Upon removal of the 100% ethanol, a 10 min 1:1 treatment with 100% ethanol and hexamethyldisilazane (HMDS) was used to transition coupons to a final fixation step with pure HMDS. The final fixation phase was completed with two HMDS treatments lasting 10 min each. Following fixation, air-dried samples were mounted on an SEM pin using carbon tape adhesive discs. Each pin-mounted sample was sputter coated for 60 s with platinum using a Cressington 208 HR sputter coater (Cressington Scientific Instruments, Watford England) set at 40 mA. Platinum sputter coated coupons were imaged using a FEI Nova Nano 200 SEM (Hillsboro, Oregon). The Everhart/Thornley Detector (ETD) were used for imaging and the Nova 200 SEM was set at a 5kv accelerating voltage, and a spot size of three. SEM imaging was done on three locations, approximately 12,740 μm^2^/coupon.

### Statistical analysis

Mean bacterial log_10_ densities were calculated based on CFU counts from control and DSB coupons. Average bacterial log_10_ densities for each dry time and temperature were compared for significant statistical differences using the least squares method of Proc Glm to fit linear models (*n* = 70; α = 0.05). The interaction between each dry time and temperature were also analyzed using the same procedure. Significant differences between group means of bacterial log_10_ densities per dry time and temperature were compared pair-wise using Tukey adjustments SAS version 9.4 (SAS Institute, Cary, NC) for all statistical analysis. Multiple logistic regression analysis was used to determine the relationship between the EPS presence and mean log_10_ densities per coupon.

## Results

### *S. aureus* DSB sufficient for disinfectant efficacy testing were established after drying at 25 °C and 30 °C

**Fig. 1:**
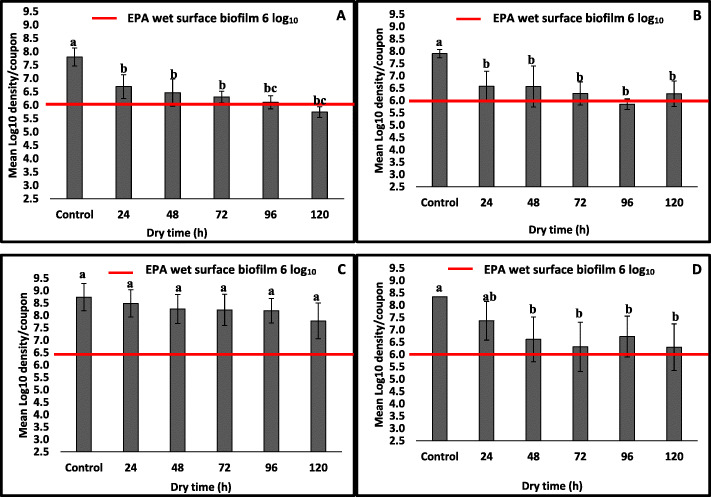
Panel **a** Mean log_10_ densities of *S. aureus* ATTC-6538 dry surface biofilms developed at 25 °C for 24–120 h Panel **b** Mean log_10_ densities of *S. aureus* ATTC-6538 dry surface biofilms developed at 30 °C for 24–120 h Panel **c** Mean log_10_ densities of *P. aeruginosa* ATTC- 15442 dry surface biofilms established at 16 °C for 24–120 h Panel** d** Mean log_10_ densities of *P. aeruginosa* ATTC-15442 dry surface biofilms established at 21 °C for 24–120 h. Bars with the same letter or pairs of Tukey letters within each panel are not statistically different

For the purpose of this study, we defined DSB as “hydrated” bacterial biofilms that remained viable despite prolonged dehydration (24 h – 120 h) under low nutrient availability and were confirmed by SEM imaging to be encased by EPS. For both drying temperatures (25 °C or 30 °C), the overall *S. aureus* mean log_10_ density per coupon ranged from 6.64 ± 0.08 (coefficient of variation; CoV =1.20%) after 24 h of drying to 6.01 ± 0.38 (CoV = 6.32%) at a dry time of 120 h. Specifically, mean log_10_ densities of 6.69 ± 0.45 (CoV = 6.73%) and 6.58 ± 0.60 (CoV = 9.12%) *S. aureus* DSB were recovered per coupon after drying for 24 h at 25 °C and 30 °C, respectively. Statistically, the bacterial mean log_10_ densities per coupon after 24 h of drying were not significantly higher than those achieved after 48 h and 72 h dry times at 25 °C or 30 °C (Fig. 1a and b) (*P* ≥ 0.05). However, mean log_10_ densities after 24 h of drying were significantly higher than those achieved after 96 h and 120 h of drying (*P* < 0.05). Overall, there were no significant differences in the average log_10_ densities of DSB per coupon between different dry times (48 h, 72 h, 96 h, and 120 h) or temperatures (25 °C and 30 °C) in our method. (*P* ≥ 0.05).

Compared to control coupons with wet surface biofilms that had an average mean log_10_ density of 7.85 ± 0.08 per coupon, and regardless of the dry time and temperature, mean log_10_ densities of dry surface *S. aureus* biofilms per coupon were significantly lower (Fig. 1a and b) (*P* < 0.05). On average, the exposure of wet surface biofilms to dehydration resulted in a 1.56 ± 0.30 log_10_ reduction per coupon.

### Higher log_10_ densities of DSB of *P. aeruginosa* were recovered at 16 °C compared to 21 °C

On average, statistically higher log_10_ densities of *P. aeruginosa* DSB were achieved after drying at 16 °C compared to 21 °C (*P* < 0.05; Fig. 1c and d). Mean log_10_ densities of DSB varied from 8.49 ± 0.55 (CoV = 6.48%) per coupon after drying at 24 h to 7.78 ± 0.72 (CoV = 9.25%) at 120 h when dried at 16 °C. However, when dried at 21 °C, the average *P. aeruginosa* log_10_ DSB density per coupon reduced to 7.37 ± 0.78 (CoV = 10.58%) at a dry time of 24 h and 6.30 ± 0.94 (CoV = 14.92%) after 120 h.

There were also statistically significant differences among dry surface *P. aeruginosa* biofilm mean log_10_ densities achieved after drying at 16 °C and 25 °C for 24 h, 48 h, 72 h, 96 h and 120 h, as well as for log_10_ densities achieved from wet surface biofilms on control coupons (*P* ≥ 0.05; Fig. 1c). When the dry temperature was set at 21 °C, no statistically higher mean log_10_ densities of DSB per coupon were achieved after 24 h of drying compared to 48 h, 72 h, 96 h and 120 h (*P* < 0.05; Fig. 1d).

### EPS were visible in *S. aureus* and *P. aeruginosa* DSB

**Fig. 2:**
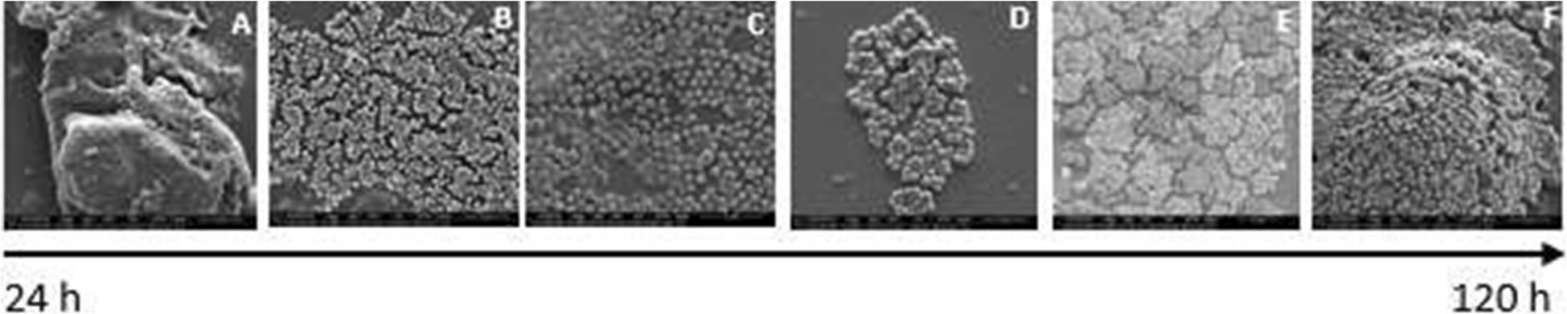
Panel** a** Scanning electron microscope (SEM) image of wet surface biofilms of *S. aureus* before drying Panel **b** dry surface biofilms of *S. aureus* developed 24 h after drying at 25 °C Panel **c** dry surface biofilms of *S. aureus* developed 48 h after drying at 25 °C Panel** d** dry surface biofilms of *S. aureus* developed 72 h after drying at 25 °C Panel** e** dry surface biofilms of *S. aureus* developed 96 h after drying at 25 °C Panel** f** dry surface biofilms of *S. aureus* developed 120 h after drying at 25 °C. Magnification: 10,000X

**Fig. 3:**
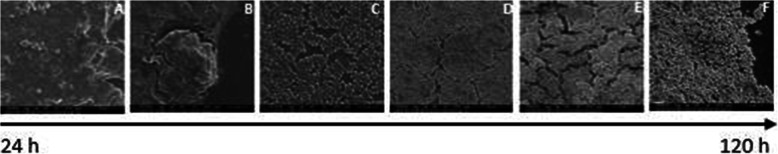
Panel** a** Scanning electron microscope (SEM) image of wet surface biofilms of *S. aureus* before drying at 30 °C Panel** b** DBS of *S. aureus* developed 24 h after drying at 30 °C Panel** c** DBS of *S. aureus* developed 48 h after drying at 30 °C Panel** d** DBS of *S. aureus* developed 72 h after drying at 30 °C Panel** e** DBS of *S. aureus* developed 96 h after drying at 30 °C Panel** f** DSB of *S. aureus* developed 120 h after drying at 25 °C. Magnification: 10,000X

*S. aureus* DSB were evaluated for EPS as previously described by Ahmatroudi et al. [20] and Ledwoch et al. [15]. The same considerations were used for *P. aeruginosa.* For the purpose of this study, coupons with EPS were those that showed cocci of *S. aureus* or rod-shaped *P. aeruginosa* encased partially or completely within a matrix as viewed with SEM. Overall, 84% of coupons (42/50) with DSB of *S. aureus* had detectable EPS. Specifically, 92% (23/25) and 76% (19/25) of all coupons with *S. aureus* DSB developed at 25 °C and 30 °C, respectively, had visible EPS through SEM imaging (Figs. 2 and 3). DSB coupons without the EPS were those with bacterial cells present but without evidence of the encasing or cross-linking matrix typical of *S. aureus* or *P. aeruginosa* biofilms. EPS was identified in only 80% (32/40) of coupons harvested between 48 h and 120 h at 25 °C and 30 °C. At 25 °C EPS was identified in 100% (15/15) of *S. aureus* DSB dried for 24 h, 48 h and 72 h (Figs. 2).

**Fig. 4:**
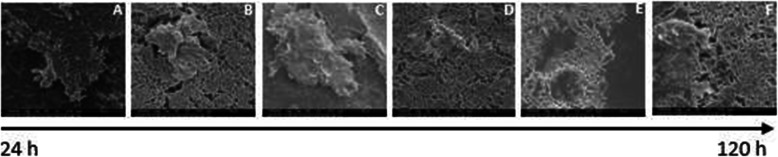
Panel** a** Scanning electron microscope (SEM) image of wet surface biofilms of *P. aeruginosa* before drying at 16 °C Panel** b** DSB of *P. aeruginosa* 24 h after drying at 16 °C Panel** c** DSB of *P. aeruginosa* 48 h after drying at 16 °C Panel** d** DSB of *P. aeruginosa* 72 h after drying at 16 °C Panel** e** DSB of *P. aeruginosa* 96 h after drying at 16 °C Panel** f** DSB of *P. aeruginosa* 120 h after drying at 16 °C. Magnification: 10,000X

**Fig. 5:**
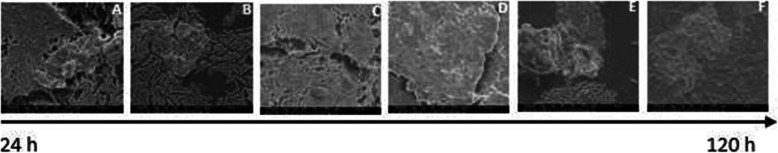
Panel** a** Scanning electron microscope (SEM) image of wet surface biofilms of *P. aeruginosa* before drying at 21 °C Panel** b** DSB of *P. aeruginosa* 24 h after drying at 21 °C Panel** c** DSB of *P. aeruginosa* 48 h after drying at 21 °C Panel** d** DSB of *P. aeruginosa* 72 h after drying at 21 °C Panel** e** DSB of *P. aeruginosa* 96 h after drying at 21 °C Panel** f** DSB of *P. aeruginosa* 120 h after drying at 21 °C. Magnification: 10,000X

EPS was also present in *P. aeruginosa* DSB (Figs. 4 and 5). On average, 76% (19/25) of all coupons dried at 16 °C had characteristic EPS regardless of the dry time. All samples (25) had EPS when dried at 21 °C. There was a notable increase in EPS on *P. aeruginosa* DSB coupons with 48–120 h of drying at 16 °C, suggesting an increase in EPS production with prolonged dehydration.

## Discussion

In this study, we developed a rapid in vitro model for establishing DSB of *S. aureus* and *P. aeruginosa*. We extensively tested wet surface biofilm dehydration endpoints and determined whether we could make a dehydrated biofilm with consistent counts above the six log_10_ required by the EPA to support wet surface biofilm disinfection claims on registered products [43]. Specifically, we tested different dry temperatures (25 °C and 30 °C for *S. aureus*, 16 °C and 21 °C for *P. aeruginosa*) and dehydration times 24 h, 48 h, 72 h, 96 h and 120 h. We found that overall, there were no statistically significant differences in the mean log_10_ densities of DSB per coupon when the drying temperature was 25 °C or 30 °C. For *P. aeruginosa* DSB developed on coupons, we found significant differences in mean log_10_ densities per coupon between drying temperatures of 16 °C or 21 °C. For *S. aureus* and *P. aeruginosa,* the average log_10_ density of DSB met and exceeded the US Environmental Protection Agency (EPA) minimum standard of six log_10_ [43] required for wet surface biofilm disinfection claims.

### A minimum six log_10_ density of DSB for disinfectant efficacy testing achieved

While there is currently no standard for testing bactericidal efficacy of disinfectants against DSB [37], we elected to use the EPA’s minimum six log_10_ standard of wet surface biofilms as our model validation standard. A six log_10_ density of DSB was achieved for *S. aureus* and *P. aeruginosa.* Our findings are similar to those of Almatroudi et al. who established an average log_10_ density of 7.13 ± 0.04 of partially dehydrated dry surface *S. aureus* per coupon using a 12-day biofilm development model [20]. Chowdhurry et al. used the same 12-day model proposed by Almatroudi et al. [20] and achieved on average 6.32 ± 0.13 log_10_ CFU of *S. aureus* DSB per coupon [44]. The rapid method we developed for *S. aureus* had similar average log_10_ densities of DSB per coupon (6.69 ± 0.45 and 6.58 ± 0.60 per coupon) after a 24 h complete desiccation step at 25 °C or 30 °C, respectively. Further desiccation from 48 to 120 h did not result in a significant decline in mean log_10_ densities of DSB per coupon, suggesting the consistency of our rapid method and the persistence of DSB of *S. aureus* on environmental surfaces under adverse conditions. In a recent review by Onyango and Alreshidi, the authors reported that *Staphylococci* could be persistent in nutrient–deficient environments in healthcare settings and contribute significantly in the incidence of HAI [45]. The previously developed 12-day model for growing partially dehydrated DSB of *S. aureus* included repeated cycles of hydration and dehydration [20]. Our rapid model though somewhat similar, involved only one 48 h hydration cycle and one subsequent dehydration cycle. However, both studies achieved similar average log_10_ densities of DSB per coupon. Once formed, DSB may stay dehydrated and may only rehydrate when surface disinfection occurs [46]. The CDC recommends daily or three times per week (approximately every 48 h) surface disinfection episodes in healthcare facilities [41]. Considering this standard, the proposed rapid model is promising for testing the efficacy of disinfectants as antimicrobials can be tested in vitro to mimic DSB disinfection every 24 h or 48 h.

We achieved higher mean log_10_ densities after dehydrating wet surface biofilms of *P. aeruginosa* for 24 h compared to *S. aureus.*. However, there were no significant reductions in the mean long_10_ densities of *P. aeruginosa* DSB per coupon with prolonged drying from 48 to 120 h. This suggests rapid maturation of *P. aeruginosa* DSB during early drying stages. Meesilp and Mesil suggested that within a two-hour period, *S. aureus* and *P. aeruginosa* inoculated on stainless steel surfaces could develop into mature biofilms [47]. As expected, lower mean log_10_ densities were achieved on all dried coupons compared to controls. This finding is similar to those of Meesilp and Mesil who suggested that although mature, the average density of biofilms on stainless steel surfaces in vitro reduced after 24 h of growth [47]. While we did not attain mean log_10_ densities per coupons expected from wet biofilm preparation following standard EPA methods (8.0–9.5 log_10_ CFU/coupon for *P. aeruginosa* and 7.5–9.0 log_10_ CFU/coupon for *S. aureus*) [38], our rapid method consistently achieved the minimum six log_10_ biofilm density required for disinfectant efficacy claims against biofilms.

### Mean log_10_ densities for DSB explained by EPS

Overall, we found a significant relationship between the mean log_10_ density of viable bacteria cells recovered from DSB of *S. aureus* and *P. aeruginosa* and the presence of EPS on sampled coupons. Specifically, we found that although relatively lower mean log_10_ CFU of DSB per coupon were recorded at near room temperatures (25 °C and 21 °C for *S. aureus* and *P. aeruginosa,* respectively), more DSB developed at these temperatures after 24 h, 48 h and 72 h of drying were encased within EPS. This possibly explains why it was more difficult to recover higher mean log_10_ CFU per coupon at these temperatures than at 16 °C and 30 °C. Previous authors reported that the EPS encases underlying bacterial cells, preventing direct contact with solutions such as disinfectants [9, 11, 12]. Similarly, in a study of multi-species DSB on surfaces collected from hospital wards, Ledwoch et al. found that *S. aureus* and *Pseudomonas* spp. were among the most prevalent pathogens on the studied surfaces, with SEM imaging confirming the presence of EPS clusters [15]. Almatroudi et al. also demonstrated that partially dehydrated DSB of *S. aureus* had multi-layered biofilm matrices similar to those on dry sterile supply boxes in a hospital [20]. These previous studies support our suggestion that at room temperature, *S. aureus* and *Pseudomonas* spp. biofilms are more likely to be encased within an EPS. Specifically, DSB survive drying through EPS production, and the expression of inherent resistance traits against environmental stress factors [48]. Under stressful conditions as starvation and dehydration, *S. aureus* and *P. aeruginosa* quickly recover in media as exposure to one form of stress may induce protection against other stress factors [49]. This has been linked to the bacterial adhesion phase in biofilm formation as adhesion induces the production of stress proteins [50, 51], which protect bacterial biofilms from adverse conditions as nutrient deprivation [52].

With regards to disinfectant efficacy testing against DSB, developing *S. aureus* and *P. aeruginosa* biofilms at near room temperatures could provide a more robust and challenging scenario for disinfectant efficacy testing and subsequent product registration with the EPA. Moreover, DSB are more tolerant to disinfection than wet surface biofilms [26, 37]. While our study successfully developed a rapid in vitro model for growing DSB, it is limited in that multi-species biofilms were not studied to better mimic the real-world use of disinfectants. We also recognize that our method involves a continuous fluid flow phase that may not be typical of the way DSB are formed on inanimate surfaces in healthcare facilities. The same could be argued for wet surface biofilms used in disinfection efficacy testing. In addition to the above stated limitations, we acknowledge that our EPS identification is limited to the areas of the coupons investigated by SEM, therefore false negatives are possible.

## Conclusion

We found that mean log_10_ densities of DSB significant for disinfectant efficacy can be developed at near room temperatures. We also found that despite prolonged drying, the bacterial density of DSB per coupon does not significantly reduce over time. Further, DSB developed at near room temperatures are predominantly encased within EPS. It is therefore critical to patient safety for the EPA to establish an efficacy method for testing disinfectants against DSB as recent studies have called into question the validity of using planktonic testing methods to represent a real world environment, where bacteria are generally encased in a biofilm on surfaces [26, 30]. As with new model development efforts, there is more to be learned about the intricacies of the DSB cell state, including stress response mechanisms and level of cross-protection under the conditions used here.

## Data Availability

All quantitative data generated or analysed during this study are included in this published article.

## Abbreviations

ATCC: American type Culture collection
CDC: Center for Disease Control
CFU: Colony forming unit
EPA: Environmental Protection Agency
EPS: Extracellular polymeric substances
HAI: Healthcare-associated infection
HMDS: Hexamethyldisilazane
PBS: Phosphate buffered saline
R2a: Reasoner’s 2a
SEM: Scanning electron microscope
TSA: Tryptic soy agar
TSB: Tryptic soy broth
ASTM: American Society for Testing and Materials
